# Conservative Management of a Prostate-Symphyseal Fistula With Osteomyelitis: A Case Report and Management of a Rare Complication

**DOI:** 10.7759/cureus.24032

**Published:** 2022-04-11

**Authors:** Gesine Peters, Joshua Winston, Fadi Nuwayhid, Owen Pointon

**Affiliations:** 1 University of Tasmania, School of Medicine, Royal Hobart Hospital, Hobart, AUS; 2 Department of Urology, Royal Hobart Hospital, Hobart, AUS; 3 Department of Medical Imaging, Royal Hobart Hospital, Hobart, AUS

**Keywords:** osteomyelitis, transurethral resection of prostate, pelvic radiation, conservative management, prostate-symphyseal fistula

## Abstract

This article presents the case of a 77-year old male who was found to have a prostate-symphyseal fistula with associated pubic symphysis osteomyelitis. He had a history of previous radiation for prostate cancer and two transurethral resections of the prostate. He was managed conservatively with long-term antibiotics and urinary diversion as he was a suboptimal surgical candidate. To our knowledge, this case report is the first reported successful conservative management of a prostate-symphyseal fistula.

## Introduction

A prostate-symphyseal fistula is a rare late complication of pelvic radiation and transurethral resections of the prostate (TURPs). There have been very few reported cases of this type of fistula in the literature [1-5], all of which were surgically managed. Our case describes a multidisciplinary approach to conservative management with the involvement of the infectious diseases, orthopedics, urology, physiotherapy, occupational therapy, and community nursing teams. The case describes the complexities of conservative management, as well as the complexities of making the initial diagnosis, as conventional imaging was not sufficient to detect the prostate-symphyseal fistula.

## Case presentation

A 77-year-old male presented to the Emergency Department with a two-day history of being unable to walk due to severe suprapubic pain and lower urinary tract symptoms (LUTS). This presentation occurred with a background of prostate cancer treated with definitive radiotherapy in 2009, followed by a TURP in 2017 for LUTS. A second TURP was performed six weeks prior to this presentation, also for LUTS. Following the latest TURP and prior to this presentation, the patient had attended the Emergency Department on two occasions and was treated for cystitis without referral to Urology at both presentations.

**Figure 1:**
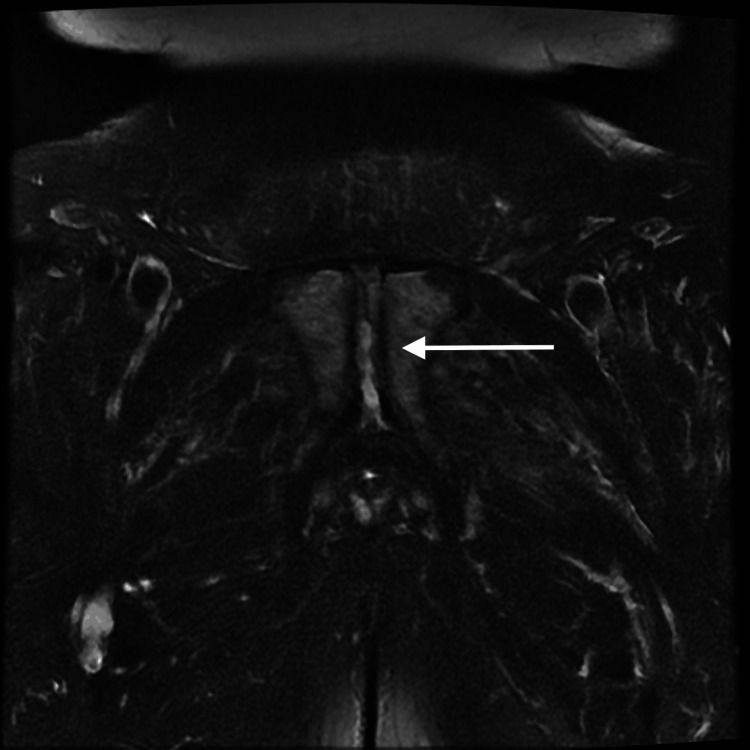
MRI at the initial presentation. The axial view shows a fistula tracking from the prostate coursing anteriorly to the symphysis pubis. There is an increased signal of the symphysis pubis, in keeping with osteomyelitis. MRI: magnetic resonance imaging

**Figure 2:**
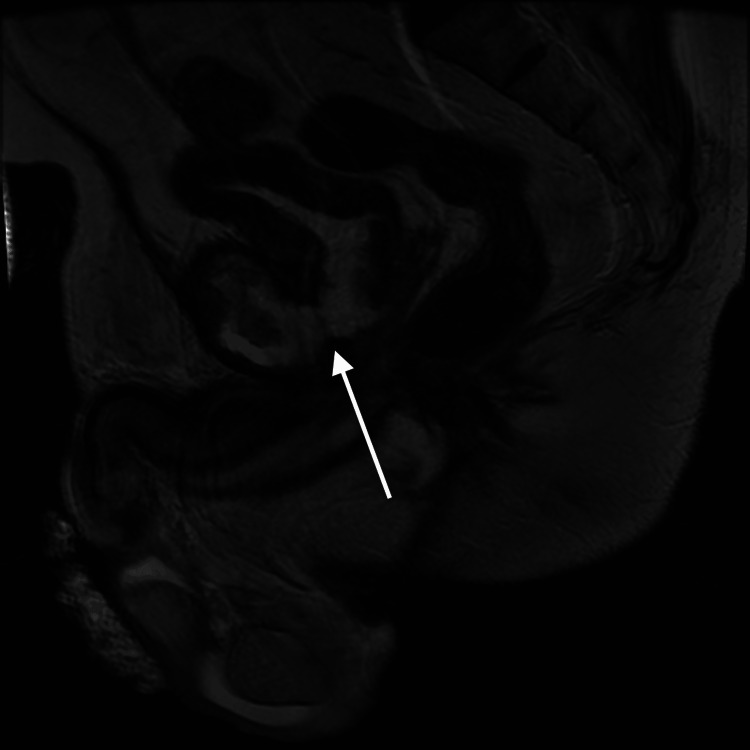
MRI at the initial presentation. The sagittal view shows a fistula tracking from the prostate coursing anteriorly to the symphysis pubis. MRI: magnetic resonance imaging

At his third presentation to the Emergency Department, a computed tomography (CT) of the abdomen suggested resolving cystitis and changes within the pubic symphysis suspicious for an infectious collection. Subsequently, magnetic resonance imaging (MRI) was ordered to better visualize pelvic organs, which confirmed a fistula between the prostate and pubic symphysis, with associated osteomyelitis of the pubic symphysis (Figures 1, 2).

Urine culture showed coagulase-negative *Staphylococcus* and *Escherichia coli*.

Orthopedics and infectious disease teams were consulted, and it was collectively decided to trial a conservative management plan as the patient was a suboptimal surgical candidate given his previous pelvic radiation. This involved insertion of an 18-F indwelling catheter and intravenous (IV) vancomycin in conjunction with oral ciprofloxacin for six weeks, as suggested by the infectious disease team.

**Figure 3:**
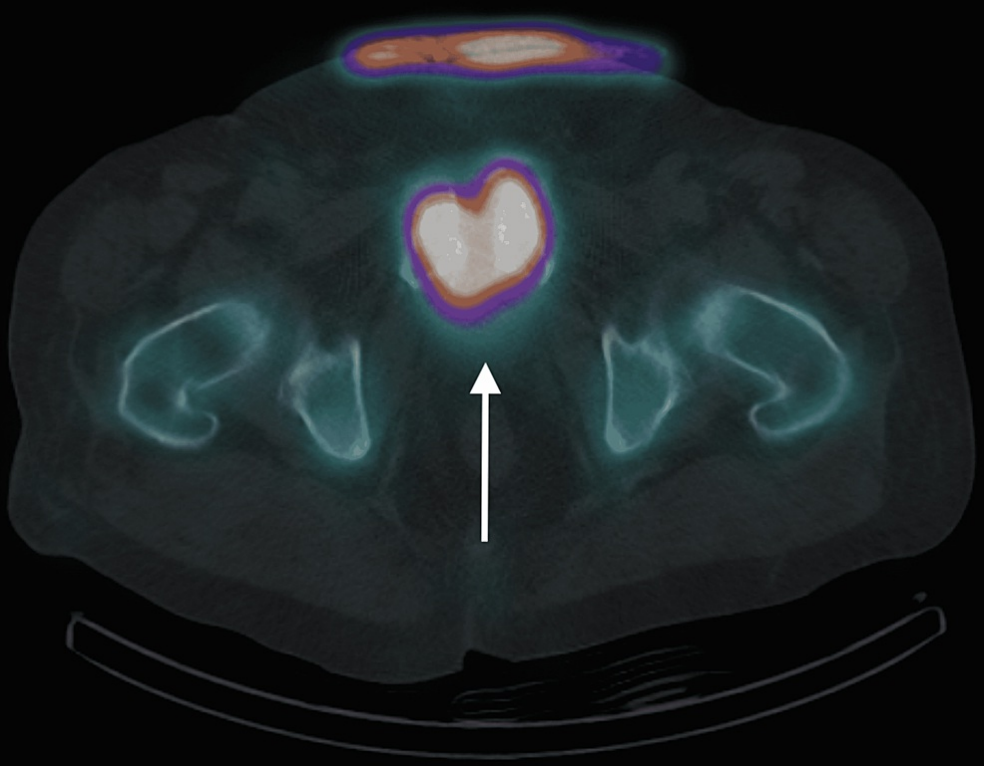
Bone scan SPECT/CT. Bone scan SPECT/CT axial image shows intense uptake in the anterior pubic bones. SPECT/CT: single-photon emission computed tomography/computed tomography

Following six weeks of conservative treatment, an interval MRI showed unchanged osteomyelitis. The case was discussed at a multidisciplinary meeting involving the infectious disease, radiology, urology, and orthopedics teams, and it was decided to exchange the indwelling catheter for a 16-F suprapubic catheter for urinary diversion and to change antibiotic treatment to oral clindamycin for a further nine weeks. After completion of this course of antibiotics, the infectious disease team requested a bone scan to visualize the extent of residual infection. The bone scan showed persisting chronic inflammation despite antibiotic therapy (Figure 3).

**Figure 4:**
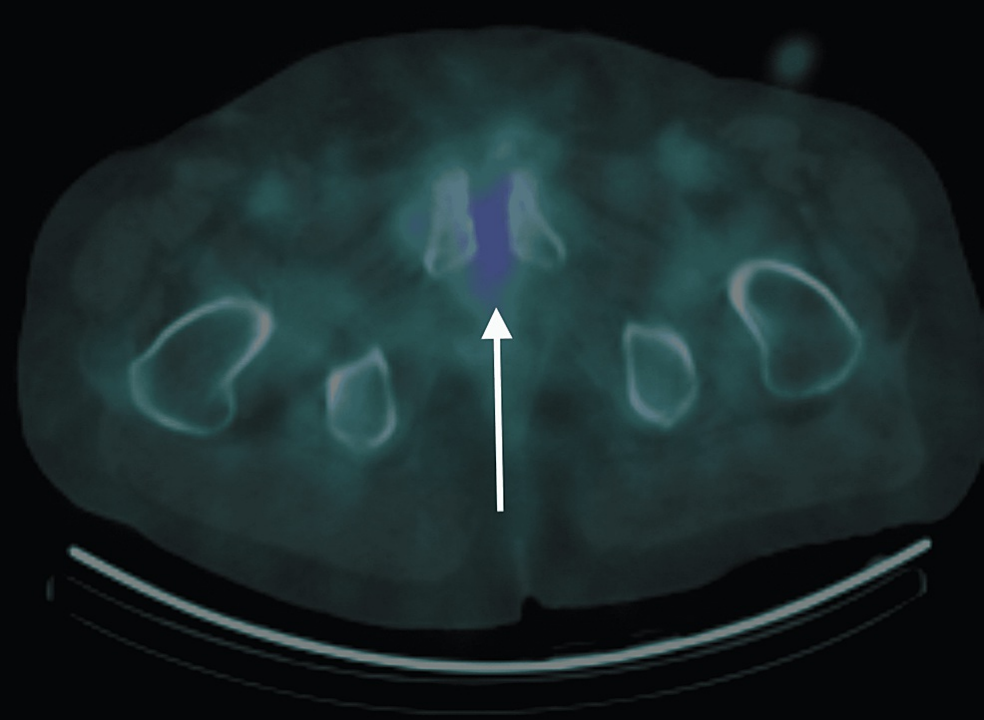
Gallium scan SPECT/CT. Axial SPECT/CT gallium scan (48 hours post-gallium 67 citrate injection) shows minimal uptake within the pubic symphysis in comparison to the bone scan done previously. This is consistent with treated osteomyelitis. SPECT/CT: single-photon emission computed tomography/computed tomography

Therefore, a gallium scan was completed three weeks after the bone scan. This more accurately shows signs of current inflammatory processes, whereas bone scans have a lag time displaying inflammatory changes despite the resolution of the infection. The gallium scan showed minimal uptake in the pubic symphysis, consistent with resolved osteomyelitis (Figure 4).

This corresponded with the resolution of the patient’s pain, allowing him to return to near full mobility without the need for further procedures.

## Discussion

Fistula formation following TURP is rare and there have been very few reported cases [1-6]. To our knowledge, this is the first paper describing this rare fistula formation between the prostate and pubic symphysis that was successfully conservatively managed, treating the infection and pain caused by this phenomenon.

Current literature suggests that fistula formation is due to damage to the anterior commissure of the prostate, which is the thinnest part of the capsule, therefore most susceptible to injury [6]. The etiology of fistula formation is usually unclear. It is suggested that patients’ previous radiation to the prostate and previous TURPs are contributing factors. Either of these two interventions or a combination of the two could have led to the fistula formation between the prostate and pubic symphysis. This, in turn, allowed chronic collection of fluid in the pubic symphysis, resulting in osteomyelitis.

There is one documented case of successful conservative management of a prostate-lymphocele sinus tract; however, this differs anatomically from this case of prostate-pubic symphysis fistula formation [6]. All other reported cases have either been managed with first-line surgical correction or have trialed conservative management that subsequently failed, leading to future surgical intervention [1-5]. In these cases, surgical management involved surgical excision and closure and was associated with high risks of morbidity and mortality [1,2,4,5].

It is important to note that this patient had presented to the Emergency Department previously with similar symptoms. He was only diagnosed with fistula formation at his third presentation, triggering a urology referral. This shows the complexity of the diagnosis due to an atypical presentation, further complicated by this fistula only being adequately visualized on MRI compared to X-ray or CT modalities. Therefore, this rare etiology of fistula formation can be easily missed or remain undiagnosed.

In complex cases such as this, it is important to take a multidisciplinary team approach to achieve the best possible outcome for the patient. Case studies have shown that when a multidisciplinary team is involved in patient care, there is an improvement in health outcomes [1,2,4,5].

## Conclusions

This case highlights the complexity of making the diagnosis of a fistula formation between the prostate and pubic symphysis. There are two main avenues of management, namely, surgical and conservative, the latter described in this case. Conservative management is a long-term treatment, and it is important to involve a multidisciplinary team in the care of patients with similar presentations for the best possible outcomes.
